# Thermal Adaptation and Diversity in Tropical Ecosystems: Evidence from Cicadas (Hemiptera, Cicadidae)

**DOI:** 10.1371/journal.pone.0029368

**Published:** 2011-12-29

**Authors:** Allen F. Sanborn, James E. Heath, Polly K. Phillips, Maxine S. Heath, Fernando G. Noriega

**Affiliations:** 1 Department of Biology, Barry University, Miami Shores, Florida, United States of America; 2 Buchanan Dam, Texas, United States of America; 3 Miramar, Florida, United States of America; 4 Department of Biological Sciences, Florida International University, Miami, Florida, United States of America; Universidade Federal do Rio de Janeiro, Brazil

## Abstract

The latitudinal gradient in species diversity is a central problem in ecology. Expeditions covering approximately 16°54′ of longitude and 21°4′ of latitude and eight Argentine phytogeographic regions provided thermal adaptation data for 64 species of cicadas. We test whether species diversity relates to the diversity of thermal environments within a habitat. There are general patterns of the thermal response values decreasing in cooler floristic provinces and decreasing maximum potential temperature within a habitat except in tropical forest ecosystems. Vertical stratification of the plant communities leads to stratification in species using specific layers of the habitat. There is a decrease in thermal tolerances in species from the understory communities in comparison to middle level or canopy fauna. The understory *Herrera umbraphila* Sanborn & Heath is the first diurnally active cicada identified as a thermoconforming species. The body temperature for activity in *H. umbraphila* is less than and significantly different from active body temperatures of all other studied species regardless of habitat affiliation. These data suggest that variability in thermal niches within the heterogeneous plant community of the tropical forest environments permits species diversification as species adapt their physiology to function more efficiently at temperatures different from their potential competitors.

## Introduction

The latitudinal gradient in species richness where diversity increases from polar latitudes towards the tropical ecosystems was first described by Forster [1] and von Humboldt [2]. It is the oldest recognized ecological relationship [3], [4] occurring in both plants and animals and in terrestrial and aquatic environments. However, the relationship is one of the central problems of ecology and biogeography and still remains fundamentally unexplained even though it is demonstrated in the fossil record for as long as 250 million years [3].

There have been multiple hypotheses proposed for the generation of the observed diversity gradient. The hypotheses include interactions of living systems (biotic hypotheses) or interactions with the environment (abiotic hypotheses). However, many of these hypotheses are contradictory. For example, one hypothesis suggests that lower competition in temperate ecosystems produces more *r*-selected species while the production of *K*-selected species in tropical ecosystems is a result of greater competition and niche separation [5]. This is in conflict with a hypothesis suggesting the greater predation rates and parasites found in tropical ecosystems lead to a reduction in competition for resources and the ability of a greater number of species to coexist [6].

Although living systems can interact to alter the species gradient at a local level, abiotic factors may also be important in generating the latitudinal species gradient. There are several hypotheses relating abiotic factors to the observed latitudinal species gradient. Included in these abiotic influences are the time hypothesis [7], which states where organisms in tropical ecosystems have had more time to diversify than in temperate ecosystems; the area hypothesis [8]–[10] suggesting that tropical ecosystems are larger and can support greater population densities resulting in fewer extinctions; the productivity hypothesis [11], [12] which proposes it is the greater energy available in tropical ecosystems that leads to the diversity; and the evolutionary speed hypothesis [13] which suggests species diversity is greater in warmer environments and environments which have remained stable for extended geologic time.

Another hypothesis for tropical species diversity is the ambient energy hypothesis [3], [14], [15] that suggests species diversity is influenced by the climate to which species are exposed, particularly temperature. In this case the greater species diversity is proposed to be a result of animals being able to spend more energy on reproduction in warmer climates. Janzen [16] proposed that adaptation to minor differences in physical characteristics of the tropical environment (including temperature) could lead to more complex communities and greater species diversity. Currie [17] also suggested thermoregulatory requirements influence species diversity in various habitats.

Measurements of ambient temperature in rainforest habitats show there is a vertical stratification in temperature [18]–[22]. The thermal environment is also more stable near the ground with greater temperature fluctuations occurring in the canopy [18]–[22]. These data suggest there are multiple thermal environments within the complex vegetation of tropical habitats that animals may exploit if they can adapt their physiology to specific thermal environments.

Measurement and analysis of thermal adaptation in cicadas began with the study of the thermal responses of the periodical cicada *Magicicada cassinii* (Fisher) by Heath [23]. Since that time, numerous studies have investigated thermal adaptation to various environments from five continents (see summary in [24], [25]–[34]). These studies have shown similar evolutionary tendencies in thermal preferences in species from similar environments that are otherwise geographically isolated illustrating that adaptation to ambient conditions is important in determining the thermal responses of cicadas [25]–[27], [32]. It has also been shown that thermal responses for individual species do not differ across as much as 7° latitude and 11° longitude in a single species [30] making the technique a reliable method to determine thermal adaptation to specific environments.

Investigation of the thermal biology of Argentine cicadas has provided an opportunity to test the influence of ambient temperature and habitat heterogeneity on cicada diversity. We were able to collect thermal response data for 64 taxa inhabiting the eight major phytogeographic regions of Argentina during the course of four expeditions. Analyses of these data show convergent and parallel evolutionary trends with respect to other temperate species [27], [32]. We investigate the thermal tolerances of the Argentine cicada species inhabiting tropical forest ecosystems and compare them with species inhabiting various habitats from similar and different latitudes to determine if temperature adaptation can influence species diversity in tropical forest ecosystems. Our data suggest that thermal diversity within the tropical ecosystem leads to an increase in species diversity as each species adapts to a specific thermal environment.

## Materials and Methods

Expeditions to study Argentine cicadas occurred during December 1981, December 1986–January 1987, January 1988, and January 1992. Twenty-two Argentine Provinces were explored in the search for specimens. The expeditions extended from 54°20′ to 71°20′ W longitude and 22°4′ to 47°55′ S latitude, including all Argentine phytogeographic regions except the Insular and Antarctic Provinces. No permits were required for the collection or experimentation of cicadas when the studies were performed. As an invertebrate animal, experimentation with cicadas is not regulated by any relevant national or international guidelines. Animals collected for laboratory experimentation were stored in a paper container with a moist paper towel over ice to cool the animals and minimize metabolism until experiments could be performed the afternoon or evening of the day of capture. Live mass was determined with a triple beam balance (O'Haus Scale Corporation, Cent-O-Gram Model CG 311) sensitive to ±5 mg.

Thermal responses (minimum flight, maximum voluntary tolerance and heat torpor temperatures) were determined following the procedures outlined by Heath [23] and Heath and Wilkin [35]. Body temperature (T_b_) was measured with a Physitemp Model BAT-12 digital thermocouple thermometer (Physitemp Instruments Inc, Clifton, NJ) and a type MT 29/1 29 gauge hypodermic microprobe copper/constantan thermocouple accurate to ±0.1°C and a time constant of 0.15 s^−1^ or a Telethermometer thermometer and a 26 gauge hypodermic thermistor probe. The thermocouple probes were calibrated with a National Institute of Standards and Technology thermometer to insure measurement accuracy. The probe was inserted midway into the dorsal mesothorax to measure deep T_b_. Specimens were handled only by the wings prior to insertion of the thermocouple to prevent conductive heat transfer with the insect. It should be reiterated that the thermal responses determined for individual species have been shown to be the same over time spans as great at 34 years and distances of 1000 km between sampled populations [30] and that habitat rather than phylogeny influences the evolution of thermal tolerances [32] so no potential bias due to sampling time or location or phylogeny of a species was introduced to the data.

Specimens were cooled to a torpid state to begin the thermal experiments. Animals were then tossed 1–2 m into the air while they warmed passively to the point where they could make a controlled flight or landing and T_b_ was measured. This minimum flight temperature represents the lowest T_b_ of fully coordinated activity. The maximum voluntary tolerance temperature is an upper thermoregulatory temperature representing a T_b_ when thermoregulation takes precedence over other behaviors [36]. The maximum voluntary tolerance or shade-seeking temperature was determined by placing specimens about 30 cm under a heat lamp. T_b_ was measured when the animal walked or flew away from the heat source. The final T_b_ measured in the laboratory was the heat torpor temperature. The heat torpor temperature was determined by heating an insect within a paper container with the heat lamp until movement stopped. This procedure is non-lethal with animals recovering motor control as they cool. The heat torpor temperature is the upper limit of activity and represents an ecologically lethal T_b_ since the animals are no longer able to avoid any continued increase in T_b_. Minimum flight temperature and heat torpor temperatures delineate the T_b_ range within which a species is fully active as cicadas are torpid outside of this T_b_ range.

T_b_ of animals active in the field was measured with the BAT-12 thermometer and type MT-29/1 microprobe used for the thermal adaptation experiments. The probe was inserted midway into the dorsal mesothorax to record deep T_b_. Captured animals were immobilized by constricting the insect net around the specimen to reduce movement prior to inserting the probe. This procedure minimizes potential heat transfer between the experimentor and the specimen. All measurements were recorded within 5 sec of the animal being captured. Ambient temperature (T_a_) was measured at a height of about 1 m in the shade after T_b_ was recorded. Operative temperature was measured with a black bulb thermometer placed at a site where a cicada had been perched prior to capture. The black bulb was constructed of an approximate 2 cm long hollow brass fishing sinker painted matte black with a copper/constantan thermocouple wire inserted in the cavity. A record of potential thermoregulatory behavior exhibited by an animal was made at the time of capture.

**Figure 1:**
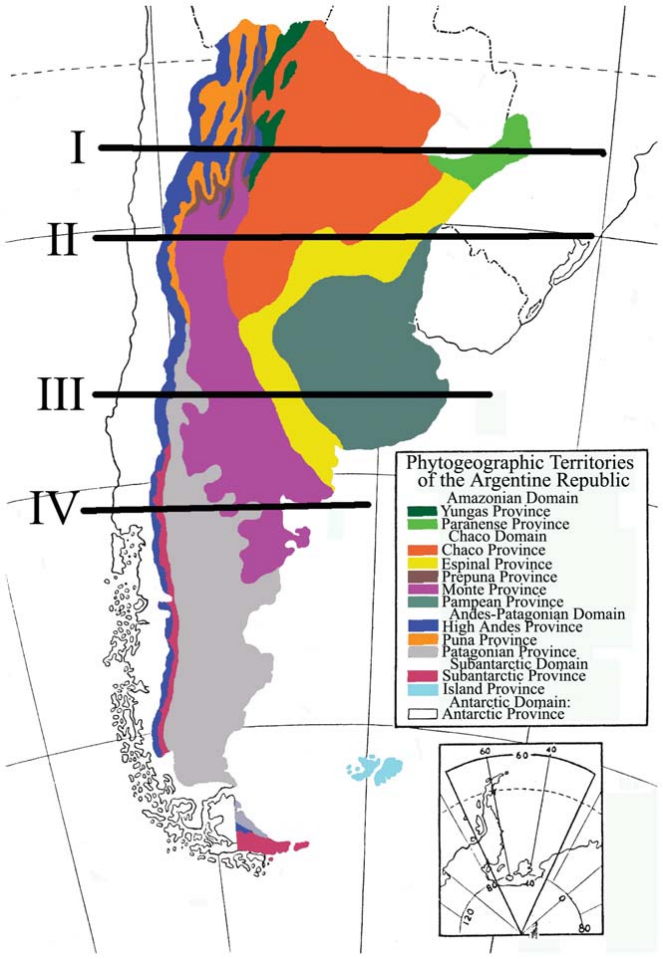
Phytogeographic provinces of Argentina (after Cabrera [**37**]). Dashed line near the top of the figure is the Tropic of Capricorn. Transects for comparative analyses of cicada thermal responses in Argentina are superimposed. Multiple plant provinces are included in each transect.

The habitat associations of cicada species were based on the phytogeography of Argentina described by Cabrera [37] (Fig. 1). There are five floristic domains and 13 provinces which are further divided into districts in this classification scheme. The domains expand over a 21°50′ latitudinal range (Argentina claims a portion of Antarctica) and a 4,500 m altitudinal range. Cicadas were assigned to individual plant provinces based on our collecting sites. Since cicada distribution is limited by phytogeography (e.g. [38]) and thermal responses for individual species are consistent across large geographic ranges [30], thermal data for the Argentine species represents mean values for all collected specimens in Argentina, not only those specimens collected on the transect lines. Some species are assigned to more than one plant province because plants, including potential host plant species, are shared by some of the plant communities [37].

Four east-west transects were made across Argentina for the comparison of thermal responses of cicadas inhabiting various habitats and latitudes (Fig. 1). Each transect incorporates various combinations of plant provinces and all provinces that support cicadas are represented. Transect I (approximately 25°S) is the most diverse with respect to plant communities. The vegetation density varies significantly across the plant communities ranging from low scrubby plants to complex tropical forest ecosystems in the Amazonian Yunga (cloud forest) and Paranense (tropical rainforest) Provinces and altitudes to 3,000 m. Transect II (approximately 30°S) contains some high elevation habitats. The vegetation is variable in density with scattered trees becoming important in the Chaco and Espinal Provinces. Transect III (approximately 33°S) includes colder high altitude habitats with sparse or low density vegetation except in the east with the addition of trees in the Espinal and the grasslands habitats of the Pampas. Transect IV (approximately 41°30′S) contains high latitude and high altitude environments characterized by sparse or low density vegetation with the exception of the trees found in the Subantarctic Province.

Statistical analyses were performed using InStat 3.0a (GraphPad Software Inc., San Diego, CA). Statistics are reported as mean ± standard deviation. Significance was taken at P<0.05. Student t-tests were performed to compare the thermal responses of individual species pairs within a single environment. Species were selected for comparative analysis because we collected the species pair in the same habitat on the same day. ANOVA analyses were performed to compare the T_b_ of groups of species active in the field and Tukey-Kramer Multiple Comparison Tests were run to determine significance between individual species pairs when the ANOVA showed statistical significance. The Mann-Whitney U-statistic was calculated for comparison of two means if data did not pass the Kolmogorov and Smirnov test.

## Results

The thermal responses of all cicada species studied are listed in Tables 1, 2, 3, 4, 5, and 6. The thermal tolerances are related to the individual environment in which a species is found. Species inhabiting lower elevation, more open desert or humid environments such as the Chaco, Espinal, Monte, and Pampas plant communities, or species calling from or near the ground, e.g. *Babras sonorivox* and *Acuticephala alipuncta*, generally exhibit elevated thermal responses while those active in understory or shaded environments or from the cooler Patagonian and Puna floristic provinces exhibit lower thermal tolerances.

**Table 1:** Thermal parameters (mean ± SD) of Argentine cicadas inhabiting the Yunga and Paranense floristic provinces.

Species	Live mass (mg)	Minimum Flight Temperature (°C)	Maximum Voluntary Tolerance Temperature (°C)	Heat Torpor Temperature (°C)	Habitat used by Species
*Herrera umbraphila* Sanborn & Heath	102±51 N = 40	18.69±2.13 N = 30	29.52±3.14 N = 36	41.41±1.31 N = 36	Yunga
*Herrera humilastrata* Sanborn & Heath	129±41 N = 19	17.19±1.56 N = 13	27.85±2.96 N = 17	39.94±1.24 N = 17	Yunga
*Odopoea insignifera* Berg	1260 N = 1	14.3 N = 1	27.7 N = 1	41.9 N = 1	Yunga
*Pachypsaltria haematodes* Torres	1460 N = 1	21.3 N = 1	30.2 N = 1	41.1 N = 1	Yunga
*Parnisa lineaviridia* Sanborn & Heath	72±27 N = 19	24.74±2.18 N = 15	33.56±3.10 N = 16	42.86±2.10 N = 16	Yunga
*Proarna insignis* Distant	434±65 N = 21	19.32±1.70 N = 18	36.42±2.46 N = 17	44.03±1.57 N = 18	Yunga
*Proarna alalonga* Sanborn & Heath	160±18 N = 4	22.13±1.16 N = 4	36.18±3.61 N = 4	42.88±0.91 N = 4	Yunga
*Proarna parva* Sanborn & Heath	95±27 N = 12	22.40±3.00 N = 10	36.19±3.48 N = 10	44.72±1.37 N = 10	Yunga
*Guyalna cuta* (Walker)	226±46 N = 13	21.59±4.18 N = 13	35.79±2.31 N = 13	46.53±2.29 N = 13	Yunga, Paranense
*Guyalna platyrhina* Sanborn & Heath	168±42 N = 12	18.24±1.75 N = 11	36.46±2.49 N = 10	44.10±1.51 N = 11	Yunga, Paranense
*Quesada gigas* (Olivier)	2505±494 N = 19	19.08±3.82 N = 17	33.77±2.91 N = 16	44.91±2.12 N = 17	Yunga, Paranense
*Calyria stigma* (Walker)	55±19 N = 11	21.25±1.14 N = 8	31.58±2.62 N = 9	40.00±1.77 N = 8	Paranense
*Carineta diardi* (Guérin-Méneville)		21.7 N = 1	33.4 N = 1	46.1 N = 1	Paranense
*Dorisiana noriegai* Sanborn & Heath	524±86 N = 13	15.60±1.19 N = 10	30.72±1.80 N = 12	45.53±0.68 N = 12	Paranense
*Fidicina torresi* Boulard & Martinelli	2345±262 N = 12	19.75±1.77 N = 10	31.97±2.74 N = 11	41.95±1.83 N = 10	Paranense
*Prasinosoma inconspicua* (Distant)	67±17 N = 12	19.76±2.16 N = 11	34.53±2.61 N = 10	44.35±1.68 N = 10	Paranense
*Zammara strepens* Amyot & Audinet-Serville	1315±205 N = 2	18.75±0.92 N = 2	27.47±1.70 N = 3	41.45±1.34 N = 2	Paranense
*Dorisiana semilata* (Walker)	388±46 N = 14	16.07±2.45 N = 15	34.85±3.61 N = 15	44.94±1.62 N = 15	Paranense
*Prasinosoma medialinea* Sanborn & Heath	101±20 N = 17	23.43±2.31 N = 16	37.93±2.58 N = 16	47.42±2.03 N = 15	Paranense

**Table 2:** Thermal parameters (mean ± SD) of cicadas inhabiting the Chaco floristic province.

Species	Live mass (mg)	Minimum Flight Temperature (°C)	Maximum Voluntary Tolerance Temperature (°C)	Heat Torpor Temperature (°C)
*Proarna parva* Sanborn & Heath	95±27 N = 12	22.40±3.00 N = 10	36.19±3.48 N = 10	44.72±1.37 N = 10
*Guyalna cuta* (Walker)	226±46 N = 13	21.59±4.18 N = 13	35.79±2.31 N = 13	46.53±2.29 N = 13
*Guyalna platyrhina* Sanborn & Heath	168±42 N = 12	18.24±1.75 N = 11	36.46±2.49 N = 10	44.10±1.51 N = 11
*Quesada gigas* (Olivier)	2505±494 N = 19	19.08±3.82 N = 17	33.77±2.91 N = 16	44.91±2.12 N = 17
*Dorisiana semilata* (Walker)	388±46 N = 14	16.07±2.45 N = 15	34.85±3.61 N = 15	44.94±1.62 N = 15
*Prasinosoma medialinea* Sanborn & Heath	101±20 N = 17	23.43±2.31 N = 16	37.93±2.58 N = 16	47.42±2.03 N = 15
*Ariasa colombiae* (Distant)	615±55 N = 6	17.47±1.01 N = 6	38.03±1.56 N = 6	46.57±1.29 N = 6
*Ariasa nigrovittata* Distant	645±121 N = 35	21.36±4.13 N = 30	37.57±2.98 N = 32	46.20±1.27 N = 32
*Mendozana antennaria* (Jacobi)	139±61 N = 17	19.57±2.65 N = 16	36.44±3.30 N = 14	47.91±1.84 N = 14
*Parnisa viridis* Sanborn & Heath	76±29 N = 28	21.98±2.58 N = 25	33.90±2.86 N = 24	42.36±1.49 N = 25
*Prasinosoma fuembuenai* Torres	85±12 N = 11	25.68±3.71 N = 10	33.11±1.34 N = 10	43.43±2.43 N = 10
*Proarna dactyliophora* Berg	139±41 N = 20	18.58±1.16 N = 19	37.71±2.73 N = 20	45.34±1.59 N = 20
*Tympanoterpes serricosta* (Germar)	175±30 N = 4	18.75±1.04 N = 4	35.40±3.79 N = 4	43.28±0.83 N = 4
*Ariasa alboapicata* (Distant)	615±86 N = 36	19.86±2.62 N = 41	36.45±3.47 N = 42	46.01±1.61 N = 40
*Ariasa arechavaletae* (Berg)	459±57 N = 8	18.51±1.54 N = 8	33.19±2.15 N = 8	44.33±0.87 N = 8
*Prasinosoma heidemanni* (Distant)	138±52 N = 23	22.55±2.36 N = 21	36.68±3.02 N = 23	45.60±2.04 N = 23
*Dorisiana drewseni* (Stål)	466±30 N = 4	17.20±1.27 N = 4	32.88±1.27 N = 4	47.75±1.19 N = 4
*Guyalna bonaerensis* (Berg)	1913±391 N = 36	16.67±1.85 N = 28	34.57±2.84 N = 27	44.93±2.29 N = 26
*Proarna bergi* (Distant)	340±74 N = 42	20.71±2.96 N = 34	37.55±2.39 N = 40	46.49±2.05 N = 39
*Acuticephala alipuncta* Torres	195±100 N = 28	20.73±2.38 N = 26	39.12±2.06 N = 27	48.48±1.23 N = 27
*Mendozana platypleura* Distant	307±85 N = 43	18.56±1.94 N = 45	37.94±2.78 N = 38	47.30±1.39 N = 38
*Chonosia septentrionala* Sanborn & Heath	1369±281 N = 6	19.32±1.24 N = 6	36.07±2.45 N = 6	46.53±2.10 N = 6

**Table 3:** Thermal parameters (mean ± SD) of cicadas inhabiting the northern Monte floristic province.

Species	Live mass (mg)	Minimum Flight Temperature (°C)	Maximum Voluntary Tolerance Temperature (°C)	Heat Torpor Temperature (°C)
*Fidicinoides ferruginosa* Sanborn & Heath	1230±232 N = 12	18.15±1.50 N = 10	34.85±2.67 N = 10	45.24±2.45 N = 8
*Proarna bufo* Distant	451±75 N = 34	19.84±1.88 N = 37	35.97±2.65 N = 40	45.38±1.96 N = 38
*Tympanoterpes cordobensis* Berg	230±53 N = 24	21.06±2.93 N = 31	37.20±2.44 N = 31	47.28±1.13 N = 32
*Mendozana platypleura* Distant	307±85 N = 43	18.56±1.94 N = 45	37.94±2.78 N = 38	47.30±1.39 N = 38
*Chonosia septentrionala* Sanborn & Heath	1369±281 N = 6	19.32±1.24 N = 6	36.07±2.45 N = 6	46.53±2.10 N = 6
*Babras sonorivox* Jacobi	107±57 N = 13	21.57±2.17 N = 13	38.60±3.48 N = 13	48.50±1.62 N = 13
*Chonosia atrodorsalis* Torres	730±211 N = 27	19.50±2.28 N = 26	34.82±3.53 N = 27	47.45±1.82 N = 27
*Chonosia crassipennis* (Walker)	1215±381 N = 37	18.48±1.74 N = 31	34.56±3.43 N = 32	44.40±1.93 N = 35
*Chonosia papa* (Berg)	1142±197 N = 9	18.76±2.90 N = 9	31.26±3.36 N = 9	47.67±2.37 N = 9
*Chonosia longiopercula* Sanborn & Heath	1386±359 N = 9	18.78±1.82 N = 9	34.22±5.00 N = 9	44.86±2.44 N = 9
*Chonosia trigonocellis* Torres	680±131 N = 3	18.23±1.95 N = 3	37.23±4.22 N = 3	44.33±2.27 N = 3
*Psephenotettix grandis* Torres	277±46 N = 6	18.18±0.89 N = 6	38.63±2.40 N = 6	48.28±1.27 N = 6
*Tettigades lebruni* Distant	350±73 N = 26	21.04±2.22 N = 32	35.39±1.94 N = 33	46.17±1.68 N = 34
*Tettigatoma maculata* Torres	877±141 N = 21	19.57±1.87 N = 19	35.05±3.17 N = 19	44.65±2.70 N = 19
*Torresia lariojaensis* Sanborn & Heath	100±30 N = 3	20.80 N = 1	36.20±1.27 N = 2	47.45±1.20 N = 2
*Torresia sanjuanensis* Sanborn & Heath	91±13 N = 10	20.75±1.58 N = 10	38.71±3.11 N = 10	49.51±1.57 N = 10

**Table 4:** Thermal parameters (mean ± SD) of cicadas inhabiting the southern Monte floristic province.

Species	Live mass (mg)	Minimum Flight Temperature (°C)	Maximum Voluntary Tolerance Temperature (°C)	Heat Torpor Temperature (°C)
*Alarcta bahiensis* (Torres)	113±20 N = 21	21.91±3.15 N = 35	37.49±2.23 N = 36	48.25±1.52 N = 35
*Alarcta minuta* (Torres)	146±38 N = 7	24.59±2.01 N = 7	37.37±1.47 N = 7	47.91±1.80 N = 7
*Alarcta micromacula* Sanborn & Heath	230±29 N = 12	21.86±2.18 N = 14	35.39±2.86 N = 14	48.67±2.16 N = 16
*Alarcta quadrimacula* Torres	244±38 N = 12	19.66±2.58 N = 10	33.03±2.82 N = 11	47.80±1.11 N = 11
*Derotettix mendosensis* Berg	95±39 N = 41	24.04±4.02 N = 45	37.20±2.77 N = 42	48.62±1.67 N = 44
*Tettigades varivenosa* Distant		22.24±1.30 N = 7	35.36±2.28 N = 8	50.29±1.53 N = 8
*Tettigades parva* Distant		19.18±1.32 N = 5	37.04±1.47 N = 5	47.66±1.64 N = 5
*Tettigades dumfresi* Distant	478±156 N = 11	18.40±2.12 N = 11	35.49±2.01 N = 11	49.14±1.67 N = 11
*Tettigades major* Torres	692±187 N = 13	15.83±2.08 N = 12	34.78±3.20 N = 12	45.28±2.45 N = 12

**Table 5:** Thermal parameters (mean ± SD) of cicadas inhabiting the Espinal floristic province.

Species	Live mass (mg)	Minimum Flight Temperature (°C)	Maximum Voluntary Tolerance Temperature (°C)	Heat Torpor Temperature (°C)
*Guyalna cuta* (Walker)	226±46 N = 13	21.59±4.18 N = 13	35.79±2.31 N = 13	46.53±2.29 N = 13
*Guyalna platyrhina* Sanborn & Heath	168±42 N = 12	18.24±1.75 N = 11	36.46±2.49 N = 10	44.10±1.51 N = 11
*Quesada gigas* (Olivier)	2505±494 N = 19	19.08±3.82 N = 17	33.77±2.91 N = 16	44.91±2.12 N = 17
*Ariasa alboapicata* (Distant)	615±86 N = 36	19.86±2.62 N = 41	36.45±3.47 N = 42	46.01±1.61 N = 40
*Ariasa arechavaletae* (Berg)	459±57 N = 8	18.51±1.54 N = 8	33.19±2.15 N = 8	44.33±0.87 N = 8
*Prasinosoma heidemanni* (Distant)	138±52 N = 23	22.55±2.36 N = 21	36.68±3.02 N = 23	45.60±2.04 N = 23
*Dorisiana drewseni* (Stål)	466±30 N = 4	17.20±1.27 N = 4	32.88±1.27 N = 4	47.75±1.19 N = 4
*Guyalna bonaerensis* (Berg)	1913±391 N = 36	16.67±1.85 N = 28	34.57±2.84 N = 27	44.93±2.29 N = 26
*Proarna bergi* (Distant)	340±74 N = 42	20.71±2.96 N = 34	37.55±2.39 N = 40	46.49±2.05 N = 39
*Alarcta minuta* (Torres)	146±38 N = 7	24.59±2.01 N = 7	37.37±1.47 N = 7	47.91±1.80 N = 7
*Tettigades parva* Distant		19.18±1.32 N = 5	37.04±1.47 N = 5	47.66±1.64 N = 5
*Alarcta bahiensis* (Torres)	113±20 N = 21	21.91±3.15 N = 35	37.49±2.23 N = 36	48.25±1.52 N = 35
*Proarna bufo* Distant	451±75 N = 34	19.84±1.88 N = 37	35.97±2.65 N = 40	45.38±1.96 N = 38
*Tympanoterpes elegans* Berg	196±46 N = 32	24.14±2.30 N = 33	36.78±2.46 N = 29	45.89±1.43 N = 38

**Table 6:** Thermal parameters (mean ± SD) of Argentine cicadas inhabiting the Pampas, Prepuña and Patagaonia floristic provinces.

Species	Live mass (mg)	Minimum Flight Temperature (°C)	Maximum Voluntary Tolerance Temperature (°C)	Heat Torpor Temperature (°C)	Habitat used by Species
*Dorisiana drewseni* (Stål)	466±30 N = 4	17.20±1.27 N = 4	32.88±1.27 N = 4	47.75±1.19 N = 4	Pampas
*Proarna bergi* (Distant)	340±74 N = 42	20.71±2.96 N = 34	37.55±2.39 N = 40	46.49±2.05 N = 39	Pampas
*Proarna montevidensis* Berg	335±76 N = 34	20.33±1.27 N = 21	38.78±2.24 N = 21	47.47±0.88 N = 25	Pampas
*Acuticephala alipuncta* Torres	195±100 N = 28	20.73±2.38 N = 26	39.12±2.06 N = 27	48.48±1.23 N = 27	Pampas
*Alarcta bahiensis* (Torres)	113±20 N = 21	21.91±3.15 N = 35	37.49±2.23 N = 36	48.25±1.52 N = 35	Pampas
*Proarna bufo* Distant	451±75 N = 34	19.84±1.88 N = 37	35.97±2.65 N = 40	45.38±1.96 N = 38	Pampas
*Tympanoterpes elegans* Berg	196±46 N = 32	24.14±2.30 N = 33	36.78±2.46 N = 29	45.89±1.43 N = 38	Pampas
*Psephenotettix grandis* Torres	277±46 N = 6	18.18±0.89 N = 6	38.63±2.40 N = 6	48.28±1.27 N = 6	Prepuña
*Psephenotettix minor* Torres	225±77 N = 11	19.29±1.77 N = 11	37.09±1.93 N = 10	49.01±1.67 N = 11	Prepuña
*Tettigades dumfresi* Distant	478±156 N = 11	18.40±2.12 N = 11	35.49±2.01 N = 11	49.14±1.67 N = 11	Patagonian
*Tettigades lebruni* Distant	350±73 N = 26	21.04±2.22 N = 32	35.39±1.94 N = 33	46.17±1.68 N = 34	Patagonian
*Tettigades major* Torres	692±187 N = 13	15.83±2.08 N = 12	34.78±3.20 N = 12	45.28±2.45 N = 12	Patagonian
*Alarcta blanchardi* (Torres)	80 N = 1	23.50±1.64 N = 8	37.04±2.41 N = 9	48.33±1.48 N = 9	Patagonian
*Tettigades bosqi* Torres	490±240 N = 2	18.15±0.78 N = 2	38.10±1.70 N = 2	48.55±0.07 N = 2	Patagonian
*Tettigades sarcinatrix* Torres	320 N = 1	21.93±1.45 N = 7	37.08±2.20 N = 8	48.24±1.54 N = 8	Patagonian

The behavior of a species can influence the absolute values of thermal tolerances for that a species within a given environment. For example, *Psephenotettix grandis* exhibits a rare acoustic strategy for cicadas, its calling song is produced while the male is flying. The elevated thermal tolerances found in species of *Psephenotettix* is an adaptation to the greater potential heat load caused by its acoustic behavior even though it inhabits an elevated environment. The animals fly for extended periods of time searching for potential mates. The muscle activity for flight generates heat and could inhibit activity [35] and reduce potential interaction with mates if it were not for elevated thermal tolerances exhibited by these species. In contrast, the endothermic *Guyalna bonaerensis* [39] has a reduced maximum voluntary tolerance temperature in a low altitude environment but this is a strategy to save energy when elevating T_b_ with metabolic heat and increase survival time and potential interaction with mates.

**Figure 2:**
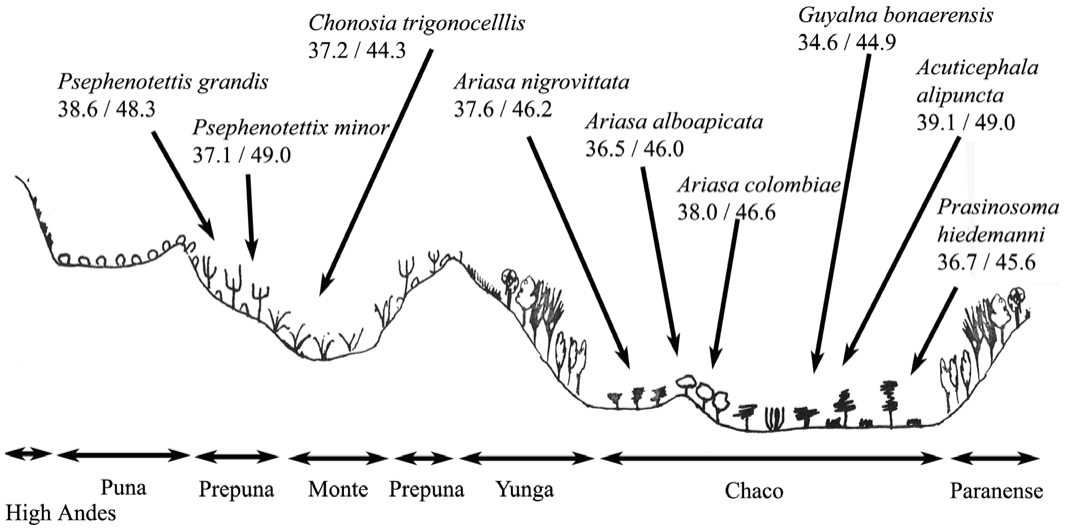
Representation of transect I illustrating the habitats and thermal responses of species inhabiting all provinces. The tropical forest (Paranense) and cloud forest (Yunga) habitats are expanded in Fig. 3. Elevated thermal responses are generally found in lower altitude and more open environments (see text for exceptions).

The cicada species inhabiting communities along the entirety of transect I exhibit elevated thermal responses in open, low altitude environments (Fig. 2). Behavioral influences on thermal responses can also be seen in some examples of this transect as outlined above. The highest thermal tolerances are found in species inhabiting the low elevation and potentially humid Chaco floristic province.

**Figure 3:**
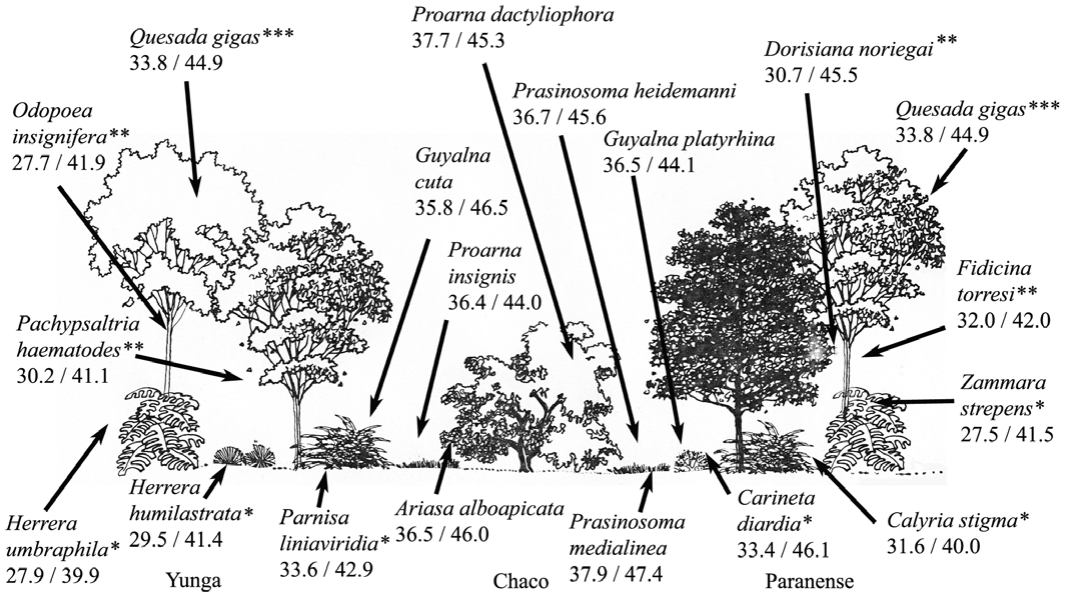
Representation of transect I between the tropical forest of the east (Paranense) and the cloud forest (Yunga) along the eastern side of the cordillera which are separated by the tropical thornscrub (Chaco) and thermal responses of representative species inhabiting all provinces. There is a greater diversity of thermal responses within the tropical forest ecosystems where there is a vertical stratification of the vegetation. Species inhabiting the understory (*), intermediate strata (**) and canopy (***) of the Yunga and Paranense are identified to facilitate comparisons of their thermal responses and to illustrate the vertical stratification of thermal responses. Thermal tolerances decrease from canopy inhabiting species to understory inhabiting species. Species in the transitional zones (e.g. *Guyalna cuta* and *Proarna insignis*) are able to use the sun and have more elevated thermal tolerances. The species inhabiting the transitional zones between the tropical forests and the Chaco and those species inhabiting the Chaco are all exposed to greater diurnal temperature variation and have more elevated thermal tolerances.

An expanded view of part of transect I illustrates the diversity of thermal responses within the stratified environments of the tropical forests (Fig. 3). The tropical forest environments of the Yunga and Paranense, which have a vertical stratification of plants, are separated by the drier and more open Chaco habitat in the illustration. There is a greater diversity of thermal responses in species inhabiting the tropical forest environments. Species inhabiting lower and middle strata within the forests have lower thermal tolerances than the species inhabiting the canopy (Fig. 3) even with the model species for the canopy, *Quesada gigas*, being an endothermic species with reduced thermal responses [40]. The canopy endotherm (*Q. gigas*) has a more elevated thermal tolerance than a sympatric middle level endotherm (*F. torresi*) so the differences are retained in individuals of similar thermoregulatory strategies as well as those individuals of differing thermoregulatory strategies (i.e. endothermy or ectothermy). Species inhabiting the more exposed areas of the forest edges, e.g. *Carineta diardia* and *Parnisa liniaviridia*, have more elevated thermal responses than those species which are found only in the deep understory environment, e.g. *Calyria stigma* and *Herrera humilastrata*. Species inhabiting the transitional zones and grasslands between the tropical forests and Chaco environments, e.g. *Guyalna cuta*, *Guyalna platyrhina*, *Proarna insignis*, *Prasinosoma medialinea*, and *Prasinosoma heidemanni*, have more elevated thermal tolerances due to the more exposed habitat and ability to use the sun to regulate T_b_. The diversity of thermal habitats within the vertical stratification has selected for species adapted to the individual thermal regimes. The diversity of cicada thermal responses in these habitats demonstrates that thermal niche separation can increase the species diversity within a tropical forest ecosystem.

Comparative analyses of the thermal responses between species in the canopy, middle levels and the understory show significant differences in both the Paranense and Yunga environments. The maximum voluntary tolerance temperature (which is a measure of the upper active T_b_) of the Paranense understory inhabiting *Zammara strepens* is the lowest value and significantly different from the middle stratum inhabiting *Fidicina torresi* (t = 2.662, d.f. = 12, P = 0.0207) and the canopy inhabiting *Quesada gigas* (t = 3.583, d.f. = 17, P = 0.0023). The same is true in the Yunga with *Q. gigas* and the understory inhabiting *Herrera umbraphila* (t = 4.605, d.f. = 50, P<0.0001) and *H. humilastrata* (t = 5.789, d.f. = 31, P<0.0001). Similar differences are seen between the canopy dwelling *Q. gigas* and the middle level species *Dorisiana noriegai* (t = 3.193, d.f. = 0.0037) and the canopy dwelling *D. semilata* and *F. torresi* (t = 2.215, d.f. = 24, P = 0.0365) in the Paranense. The understory species are also less thermal tolerant than middle and upper strata species. The heat torpor temperatures of the Paranense understory species *Calyria stigma* are less than and significantly different from the middle level *D. noriegai* (t = 9.889, d.f. = 18, P<0.0001) and the canopy inhabiting *Q. gigas* (t = 5.670, d.f. = 23, P<0.0001) as are the Yunga species *Q. gigas* and *H. umbraphila* (t = 7.393, d.f. = 51, P<0.0001) and *H. humilatrata* (t = 8.344, d.f. = 32, P<0.0001). The same is true with respect to middle level and canopy species as illustrated in the Paranense by *D. semilata* and *F. torresi* (t = 4.295, d.f. = 23, P = 0.0003) and *Q. gigas* and *F. torresi* (t = 3.676, d.f. = 25, P = 0.0011). The small sample sizes of middle level taxa collected in the Yunga prevent comparisons to the understory and canopy species from the Yunga but the absolute values of the thermal tolerances are intermediate to the canopy and understory species as seen in the Paranense species.

**Figure 4:**
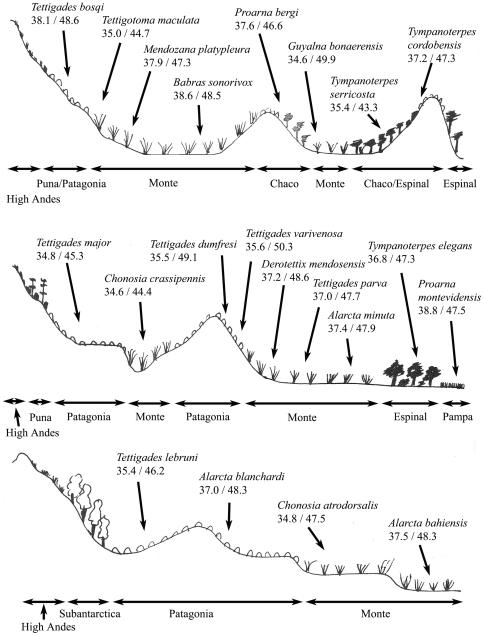
Representation of transects II (top), III (middle), and IV (bottom). The habitats and thermal responses for representative species are illustrated. Thermal responses are adapted to specific habitats and microhabitats. There is no vertical stratification of thermal responses within a given habitat as seen in the tropical forest ecosystems.

Species inhabiting plant communities along transect II (Fig. 4 top) show similar patterns of thermal tolerances as seen in transect I. Species inhabiting lower altitude, the high humidity eastern environments and more open environments exhibit elevated thermal tolerances but individual values can again be influenced by the behavior and microhabitat used by a species. For example, *Guyalna bonaerensis* and *Proarna bergi* are endothermic species [39], [40] which decreases their thermal tolerances. The elevated thermal tolerances of *Babras sonorivox* are probably an adaptation to singing from the ground where it is exposed to the higher T_a_ of the boundary layer while the reduced values for *P. bergi* and *Tympanoterpes serricosta* may be adaptations to the thick grass that the species use as host plants which would provide a thermal buffer against extremes in T_a_. The thermal responses of cicadas in transect III show a pattern of decreased thermal tolerances at higher altitude and in the cooler Patagonian and Puna floristic provinces (Fig. 4 middle). However, the thermal tolerances once again increase in species inhabiting the warm, humid environments of the east. Transect IV shows similar thermal responses across all habitats due to the similar potential thermal maximum in each habitat (Fig. 4 bottom). There is a decrease in thermal tolerance with increasing altitude except *Chonosia atrodorsalis* but members of the genus *Chonosia* exhibit lower thermal responses in all transects and appears to be characteristic of the genus (Table 3).

Comparative analyses of the thermal responses of species from the remaining transects show adaptation to particular environments but the vertical stratification seen in the tropical forests is missing. For example, in the Chaco the maximum voluntary tolerance temperature of *Ariasa nigrovittata* does not differ significantly from *A. alboapicata* (t = 1.461, d.f. = 72, P = 0.1485) or *A. columbiae* (t = 0.3659, d.f. = 36, P = 0.7166), *A. alboapicata* from *Proarna dactyliophora* (t = 1.425, d.f. = 60, P = 0.1593), *Prasinosoma heidemanni* (t = 0.2671, d.f. = 63, P = 0.7903), or *Mendozana antennaria* (t = 0.009448, d.f. = 59, P = 0.9925). The same is true in the Monte with *Tympanoterpes cordubensis* and *Proarna bergi* (t = 0.6065, d.f. = 69, P = 0.5462), *Fidicinoides ferruginosa* and *Guyalna bonaerensis* (t = 0.2704, d.f. = 35, P = 0.7884) and *Q. gigas* (t = 0.9492, d.f. = 24, P = 0.3520), *Tettigatoma maculata* and *Tettigades major* (t = 0.2302, d.f. = 29, P = 0.8196), *Alarcta micromaculata* (t = 0.3171, d.f. = 31, P = 0.7533), *Alarcta micromacula* (t = 1.748, d.f. = 28, P = 0.0914), in the Espinal with *Tympanoterpes elegans* and *Ariasa alboapicata* (t = 0.4409, d.f. = 69, P = 0.6607) and *G. bonaerensis* and *Q. gigas* (t = 0.8848, d.f. = 41, P = 0.3814), and in the Patagonian floristic province with *Tettigades major* and *Tettigades lebruni* (t = 0.7772, d.f. = 43, P = 0.4413) and *Alarcta blanchardi* (t = 1.771, d.f. = 19, P = 0.4413). The similarities continue as the heat torpor temperatures do not differ in the Chaco *Ariasa nigrovittata* and *A. alboapicata* (t = 0.5453, d.f. = 70, P = 0.5873) or *A. columbiae* (t = 0.6534, d.f. = 36, P = 0.5176), *A. alboapicata* from *Proarna dactyliophora* (t = 1.5264, d.f. = 58, P = 0.1325), in the Monte with *Tympanoterpes cordubensis* and *Proarna bergi* (t = 1.949, d.f. = 69, P = 0.0554), *Fidicinoides ferruginosa* and *Guyalna bonaerensis* (t = 0.3297, d.f. = 32, P = 0.7438) and *Q. gigas* (t = 0.3458, d.f. = 23, P = 0.7326), *Tettigatoma maculata* and *Tettigades major* (t = 0.6551, d.f. = 29, P = 0.5176), in the Espinal with *Tympanoterpes elegans* and *Ariasa alboapicata* (t = 0.3474, d.f. = 76, P = 0.7293) and *G. bonaerensis* and *Q. gigas* (t = 0.02882, d.f. = 41, P = 0.9772), and in the Patagonian floristic province with *Tettigades major* and *Tettigades lebruni* (t = 1.394, d.f. = 44, P = 0.1704).

Table 7 summarizes the T_b_ measurements of animals actively calling in the field. There is a statistically significant relationship when comparing all species (ANOVA F = 44.470, d.f. = 12, 287, p<0.0001). The mean value of the understory inhabiting species *Herrera umbraphila* is the lowest reported value and is significantly different than every other species measured (Tukey-Kramer Multiple Comparisons Test *Alarcta bahiensis* q = 18.412, p<0.001; *Alarcta quadrimacula* q = 18.542, p<0.001; *Ariasa nigrovittata* q = 12.074, p<0.001; *Chonosia crassipennis* q = 12.862, p<0.001; *Guyalna bonaerensis* q = 24.601, p<0.001; *Mendozana platypleura* q = 8.680, p<0.001; *Proarna bergi* q = 15.40, p<0.001; *Proarna insignis* q = 15.776, p<0.001; *Proarna monetvidensis* q = 17.897, p<0.001, *Quesada gigas* q = 13.974, p<0.001; *Tettigatoma maculata* q = 20.210, p<0.001; *Tympanoterpes elegans* q = 16.928, p<0.001). The other couples that showed statistical significance were *Alarcta bahiensis* and *G. bonaerensis* (q = 6.985, p<0.001), *Alarcta bahiensis* and *Q. gigas* (q = 5.534, p<0.01) and *Proarna montevidensis* and *G. bonaerensis* (q = 5.971, p<0.01),

**Table 7:** Body temperature of active animals and their habitat association.

Species	Body temperature (°C)*	Habitat
*Herrera umbraphila*	27.19±2.42 (N = 55)	Yunga (understory)
*Proarna insignis* †	36.25±1.52 (N = 12)	Yunga (grasslands)
*Quesada gigas* †	34.53±1.56 (N = 15)	Yunga (canopy), Espinal, Chaco, Paranense (canopy)
*Ariasa nigrovittata*	35.43±1.60 (N = 8)	Chaco
*Mendozana platypleura*	36.47±3.10 (N = 3)	Chaco, Monte
*Guyalna bonaerensis* †	34.45±3.13 (N = 116)	Chaco, Espinal, Monte
*Proarna bergi* †	35.76±1.18 (N = 13)	Chaco, Espinal, Pampas
*Alarcta bahiensis*	38.60±1.26 (N = 10)	Pampas, Espinal, Monte
*Tympanoterpes elegans*	36.60±1.51 (N = 13)	Pampas, Espinal
*Proarna montevidensis*	37.85±1.30 (N = 11)	Pampas
*Alarcta quadrimacula*	36.46±3.04 (N = 17)	Monte
*Chonosia crassipennis*	35.96±1.25 (N = 8)	Monte
*Tettigatoma maculata*	36.88±2.25 (N = 19)	Monte

The value for the understory inhabiting *Herrera umbraphila* is lower than and significantly different (p<0.001) from every other species regardless of habitat association.

*ANOVA F = 44.470, d.f. = 12, 287, p<0.0001.

† Endothermic species [39], [40].

A regression analysis of T_b_ as a function of T_a_ for the understory inhabiting *Herrera umbraphila* (Y = −1.644+1.154x, r^2^ = 0.8303) shows that T_b_ is similar to T_a_. The 95% confidence interval for the slope is 1.010 to 1.298. In contrast, the regression for *Guyalna bonaerensis* (Y = −21. 84+0.459x, r^2^ = 0.2704) shows that T_b_ is regulated at a level different from T_a_. A slope of a regression significantly different from one or not significantly different from zero suggests thermoregulation is occurring [41].

The T_b_ measurements of *H. umbraphila* were similar to the operative temperature measured at their site of activity suggesting the animals were thermoconforming. Mean T_b_ of active *H. umbraphila* (31.19±0.68°C, N = 7) is within the range of operative temperatures measured at the beginning and end of the measurement session in locations where the cicadas were active (30.0–31.2°C). Moving the black bulb thermometer to full sun increased the operative temperature to 35.3°C so the cicadas could have found microclimates that would increase T_b_ to higher levels when the sun was unobstructed. As the environment changed from sunny skies to fully overcast, T_b_ decreased (29.46±1.44°C, N = 8) as the operative temperature decreased to 28.2°C. T_a_ decreased slightly from 27.7°C under sunny skies to 27.3–27.0°C during overcast conditions. The change in T_b_ was strongly correlated with the changing operative temperature suggesting further that *H. umbraphila* is thermoconforming.

Mean T_b_ (27.19±2.44°C, N = 55) and mean T_a_ (24.99±1.91°C, N = 55) for *Herrera umbraphila* are statistically significant (Mann-Whitney U-statistic = 744.50, U′ = 2280.5, p<0.0001 because the standard deviations were not equal [F = 1.604, p = 0.0428] and T_a_ data did not pass the Kolmogorov and Smirnov test [KS = 0.2929, p = 0.0002]) but this difference may be a result of the sample sites which were often on disturbed forest edges to permit access to the habitat. Mean T_b_ (34.45±3.13°C, N = 116) and mean T_a_ (28.27±3.55°C, N = 116) for the thermoregulating *Guyalna bonaerensis* are statistically significant (Mann-Whitney U-statistic = 1237.0, U′ = 12219, p<0.0001 because T_a_ data did not pass the Kolmogorov and Smirnov test [KS = 0.2354, p<0.0001]).

## Discussion

The thermal tolerances of Argentine cicadas illustrate several patterns. The minimum flight temperature is related to the aerodynamics of the flight system [42]. The maximum voluntary tolerance and heat torpor temperatures are related to the habitat and behavior of the species. The maximum voluntary tolerance temperature is related to the temperature of the environment, the T_b_ at which a species is active, and the thermoregulatory behavior of the species. Species inhabiting higher altitudes and higher latitudes have lower maximum voluntary tolerance temperatures. There are lower values for the maximum voluntary tolerance if the species is found in cooler microhabitats within more complex plant communities (e.g. *Herrera umbraphila*) or is endothermic (e.g. *Guyalna bonaerensis* [40]). The vertical stratification of the Yunga and Paranense Provinces leads to differences in maximum voluntary tolerance temperatures for the species inhabiting different strata of these environments. The heat torpor temperature is related to the potential maximum thermal stress of the environment. The understory inhabiting *H. umbraphila* (39.94°C) has a significantly lower (t = 19.42, d.f. = 59, p<0.001) heat torpor temperature than the similarly sized *Derotettix mendosensis* Berg (48.62°C) that inhabits desert salt flats [27]. Habitat and elevation have significant influence on the evolution of thermal tolerances [25]–[28], [32], [33] and species of the Argentine cicada fauna exhibit many of the same trends.

We found a significantly greater diversity of the thermal preferences for the species that inhabit the floristically diverse tropical environments (Fig. 3), the Amazonian Provinces Yunga and Paranense, that extend into northern Argentina along the eastern side of the Andes and into the northeastern region of the country respectively [40]. The Yunga is characterized as cloud forest while the Paranense is a more traditional rainforest environment. Both are characterized by a vertical stratification of the various plant species [40] which generate layers of potential host plants and activity sites for cicadas and other animal species as well as decreased access to solar radiation the further from the canopy an individual is located.

The thermal responses of the cicadas inhabiting the Yunga and Paranense showed a divergence from species inhabiting other environments. We found a stratification of thermal responses between the cicada species in different vegetation layers that mirror the stratification in T_a_ that is found within tropical forest environments [18]–[22]. We found a decrease in the value of the thermal preferences as well as a decrease in total T_b_ range for activity (Table 1, Fig. 3) in animals that inhabit the areas closest to the ground, intermediate values for those species using the middle vegetation strata, and highest tolerances in those species which use the canopy for activity. The lower T_a_ in the lower level strata is occupied by animals that can function at lower T_b_. Similarly, since these understory animals experience smaller diurnal temperature changes they are not challenged nor selected to survive over a wider range of T_b_. Although temperature habitats also exhibit thermal microclimate heterogeneity (e.g. [43]), the thermal responses of species from non-tropical ecosystems do not show the same stratification of thermal responses exhibited by the tropical forest habitat species.

There are multiple examples of diverse insect groups that exhibit a stratification of species and populations within tropical ecosystems (e.g. [44]–[51]). Microclimate selection has also been suggested to influence the stratification of insects within tropical forests (e.g. [47], [48], [50]). Our data have shown that thermal adaptation to specific microclimates may provide a mechanism for the greater species diversity in the thermally diverse tropical forest ecosystems and help to explain the stratification of species within tropical forests.

**Figure 5:**
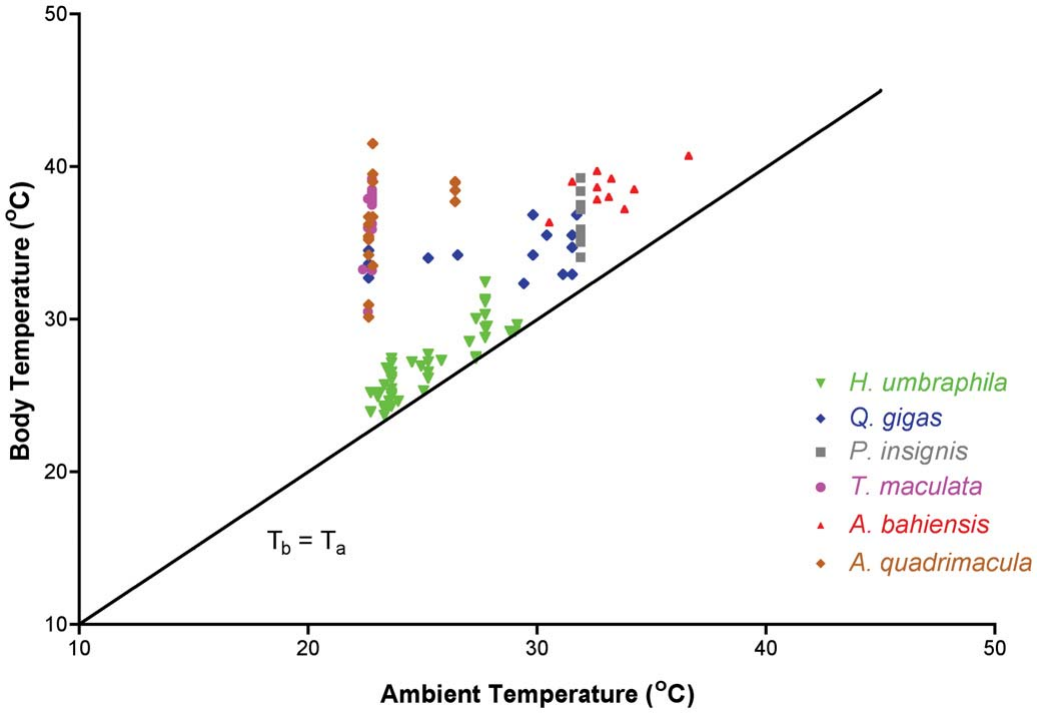
Illustration of body temperature as a function of ambient temperature in Argentine cicadas from various environments. The species from the lowest stratum of the Yunga (*Herrera umbraphila*) is significantly lower than the species which inhabit more open environments or a more elevated stratum.

The decreased thermal responses were also coupled with endothermy in some species as a mechanism to inhabit portions of the habitat that might not otherwise have been available to the species [28], [39], [40], [52]. However, endothermy was not a requirement for species to inhabit these environments. The thermal responses suggest species have adapted to these cooler microclimates and can function well without the need to elevate T_b_ to the range seen in animals which have greater access to solar radiation (Fig. 5).

The regression analysis for *Herrera umbraphila* is the first regression of T_b_ as a function of T_a_ reported for a cicada to have a slope greater than one [28], [39], [52]–[59]. The 95% confidence interval (1.010–1.298) suggests that the slope may be closer to 1.0 and that *H. umbraphila* is thermoconforming. This is the first diurnally active thermoconforming species of cicada known. All other diurnally active or endothermic species studied are thermoregulating species [23], [28], [35], [39], [52]–[65]. The deep shade of the understory may prevent *H. umbraphila* from gaining access to solar radiation in order to elevate T_b_ significantly above T_a_ and thus regulate T_b_. Further evidence to support thermoconformation is the similarity between *H. umbraphila* T_b_ and operative temperature measured at the site of activity. We demonstrated that the cicadas could increase their T_b_ to higher levels by moving the black bulb thermometer into the sun but the mean T_b_ of active animals was almost identical to the operative temperature measured in their microenvironment. Field temperatures for *H. umbraphila* are less than and significantly different from every other species examined. The T_b_ distribution of *H. umbraphila* is similar to thermoconforming lizards found in deep shade environments without access to solar radiation (e.g. [64]–[70]).

The vertical stratification of temperature within tropical forests has been shown multiple times [18]–[22]. Although in our studies we did not perform a detailed analysis of the thermal niches within the Argentine tropical forests, indirect evidence to support that Argentine tropical forests present the same type of vertical temperature stratification comes from the air temperatures measured when collecting field temperatures of active animals (Fig. 5). These temperatures were the lowest in the understory of the tropical forest even when compared to higher latitude or more elevated environments. Operative temperatures showed that animals from the understory could have elevated T_b_ further by selecting a different microclimate. Additionally, the species from the understory had significantly lower T_b_ than any other species measured including animals inhabiting higher strata within the same forests, and there were statistically significant differences between the thermal responses of animals inhabiting the different strata in the tropical forests that were not observed in species inhabiting other environments. The thermal responses of cicadas are adaptations to particular environmental conditions (e.g. [26]–[27], [32]–[33]) that are independent of phylogeny (e.g. [32]), collection site or year determined (e.g. [30]). Thermal responses are demonstrative of active T_b_ ranges of cicadas (e.g. [23], [28], [35]–[36], [39]–[40], [53]–[58], [60]–[61], [65]) and the thermal responses determined for species inhabiting the tropical forests mirror the thermal gradient measured in other tropical forest environments. These data strongly suggest that the thermal diversity of the Argentine tropical forests is similar to that documented in other tropical forests and the cicadas are adapting to particular thermal regimes within the forest.

The unique aspect of the thermal responses of Argentine cicadas is the diversity found within the tropical forest ecosystems. The thermal responses of species inhabiting the remaining habitats are similar to those determined for species in temperate habitats in North America, Africa, Europe and Australia (e.g. [25], [27], [32], [33]). This is not surprising as the influence of habitat on the thermal responses of cicadas has been used as a model to illustrate convergent evolutionary patterns in cicadas from Argentina and the United States [27] as well as within Mediterranean habitats on four continents [32].

Rather than being independent variables, we suggest that habitat heterogeneity and temperature are related in generating tropical biodiversity. The data we collected suggest that animals are able to adapt to particular thermal regimes within any given environment. It has been shown that single gene mutations can change the temperature preferences for insects [71]. The heterogeneous plant community in the tropical ecosystems leads to variability in the thermal niches available to individual species. It is this variability in thermal niches that permits species to radiate as they adapt their physiology to function more efficiently at temperatures that are different from potential competitors.
